# Tryptophan Metabolism in Depression: A Narrative Review with a Focus on Serotonin and Kynurenine Pathways

**DOI:** 10.3390/ijms23158493

**Published:** 2022-07-31

**Authors:** Ana Salomé Correia, Nuno Vale

**Affiliations:** 1 OncoPharma Research Group, Center for Health Technology and Services Research (CINTESIS), Rua Doutor Plácido da Costa, 4200-450 Porto, Portugal; anncorr07@gmail.com; 2 Institute of Biomedical Sciences Abel Salazar (ICBAS), University of Porto, Rua de Jorge Viterbo Ferreira 228, 4050-313 Porto, Portugal; 3 CINTESIS@RISE, Faculty of Medicine, University of Porto, Alameda Hernâni Monteiro, 4200-319 Porto, Portugal; 4 Department of Community Medicine, Health Information and Decision (MEDCIDS), Faculty of Medicine, University of Porto, Rua Doutor Plácido da Costa, 4200-450 Porto, Portugal

**Keywords:** Trp metabolism, serotonin pathway, kynurenine pathway, depression, inflammation, brain-derived neurotrophic factor, stress, microbiota

## Abstract

Depression is a common and serious disorder, characterized by symptoms like anhedonia, lack of energy, sad mood, low appetite, and sleep disturbances. This disease is very complex and not totally elucidated, in which diverse molecular and biological mechanisms are involved, such as neuroinflammation. There is a high need for the development of new therapies and gaining new insights into this disease is urgent. One important player in depression is the amino acid tryptophan. This amino acid can be metabolized in two important pathways in the context of depression: the serotonin and kynurenine pathways. These metabolic pathways of tryptophan are crucial in several processes that are linked with depression. Indeed, the maintenance of the balance of serotonin and kynurenine pathways is critical for the human physiological homeostasis. Thus, this narrative review aims to explore tryptophan metabolism (particularly in the serotonin and kynurenine pathways) in depression, starting with a global overview about these topics and ending with the focus on these pathways in neuroinflammation, stress, microbiota, and brain-derived neurotrophic factor regulation in this disease. Taken together, this information aims to clarify the metabolism of tryptophan in depression, particularly the serotonin and kynurenine pathways.

## 1. Introduction

**Figure 1:**
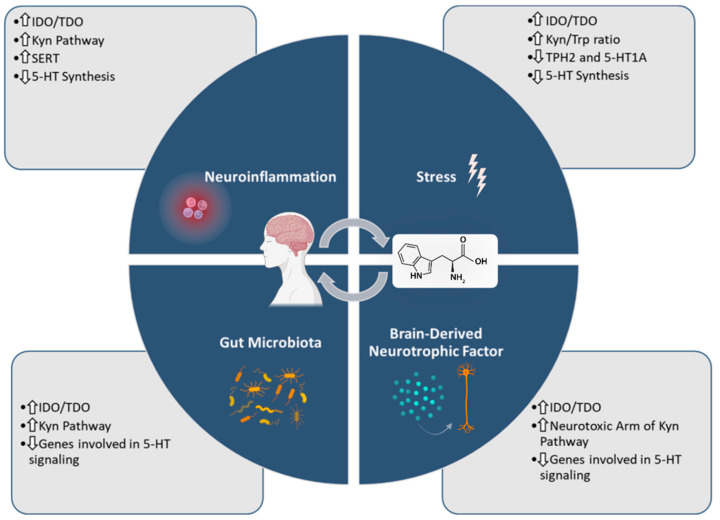
Schematic abstract of the information presented in this review. Briefly, the Trp metabolism is important in several processes connected with depression, particularly neuroinflammation, stress response, gut microbiota dysregulation, and brain-derived neurotrophic factor activity. Illustration created with BioRender [8]. TPH2: tryptophan hydroxylase 2; 5-HT1A: serotonin 1A receptor; IDO: indoleamine 2-3-dioxygenase 1; TDO: tryptophan 2,3-dioxygenase; SERT: serotonin transporter.

Depression is a disease that affects millions of people around the world. It is a very complicated disease, difficult to study and fully understand, being one of the biggest challenges in medicine and neurosciences. Currently, there are several therapeutic modalities for this disease, particularly antidepressant therapy and psychotherapy. However, despite being quite effective, there is still a lot of failure in this therapy, and, above all, relapse of depression is a common reality. Thus, the deepening of the molecular knowledge inherent to this disease to fill the existing gaps in the therapy is a major focus of neuroscience. In fact, several factors are associated with the development of depression, such as exposure to high levels of permanent stress; neuroinflammation; and general dysregulation of neurotransmitters, such as serotonin (5-HT), dopamine, and noradrenaline [1,2]. The metabolism of the amino acid tryptophan (Trp) has a high participation in all of these processes. This amino acid is obtained exclusively from the diet and plays a fundamental role in several physiological reactions. Its metabolism into 5-HT and kynurenine (Kyn) plays a key role in depression [3], as will be explained in detail throughout this manuscript. Thus, all of the processes associated with the metabolism of Trp into 5-HT/Kyn must be strictly regulated to not disturb its homeostatic balance. The Kyn pathway is important for several physiological processes including the generation of cell energy, inflammation, immune response, and neurotransmission. However, overactivation of this pathway is generally associated with a lower activation of the 5-HT pathway, promoting the development of mechanisms associated with depression. Furthermore, it is important to note that this pathway is divided into two major branches: neurotoxic and neuroprotective branches. In depression, there is much greater activation of the neurotoxic branch of this pathway [4,5]. Regarding the 5-HT pathway, it also assumes vital functions throughout the body, involving various physiological processes such as mood, motor control, regulation of circadian rhythm, and gastrointestinal regulation. In depression, this pathway is widely studied, and in general, it is known that increased levels of 5-HT and disruptions to its receptors and pathways are also widely associated with this disease [6,7]. Thus, the study of Trp metabolism in Kyn and 5-HT takes a central importance in the investigation of depression. Therefore, this narrative review will focus on these pathways in processes such as neuroinflammation, stress, microbiota, and brain-derived neurotrophic factor dysregulation in depression. The main goal is to clarify these processes, which may help in the development of new therapies or biomarkers and help to improve the quality of life of patients who suffer from this condition that is so disturbing and challenging to their daily lives. For the research, collection of papers was carried out through a search on the PubMed database, considering mainly literature published in the last 5 years and focusing on the most relevant papers for the topic. Figure 1 summarizes the information presented in this review.

## 2. Depression—A Brief Contextualization

**Figure 2:**
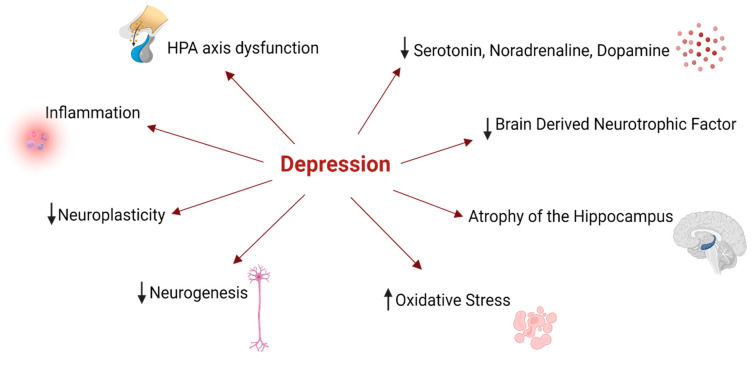
Depression is a complex disease. Hypothalamic–pituitary–adrenal (HPA) axis dysfunction, low levels of neurotransmitters (such as 5-HT, noradrenaline, and dopamine) and neurotrophic factors (such as BDNF), brain atrophy of regions such as the hippocampus, increased levels of inflammation and oxidative stress, and decreased levels of neurogenesis are some underlying features of depression. Illustration created with BioRender [8].

Depression is a globally prevalent disease. About 280 million people in the world have depression [9]. This is a very debilitating illness, characterized by anhedonia, sad mood, disrupted circadian cycle, changes in appetite or weight, lack of energy, and cognitive abnormalities. In severe cases, this disease can even lead to death by suicide [9,10]. One of the major problems associated with depression is the high rate of recurrence, therapy failure, and lack of diagnosis/treatment, especially in low- and middle-income countries [1,2,9]. However, there are effective treatment modalities, particularly psychotherapy and/or antidepressant medication [2]. Despite all of the recent advances in neurosciences, there is still no explanation for all of the molecular aspects of depression. However, it is known that biogenic amine deficiency (particularly 5-HT, dopamine, and noradrenaline), neurotrophic factors such as brain-derived neurotrophic factor (BDNF), gut microbiota deregulation, genetic, immunologic, endocrine, environmental factors, and neurogenic problems are underlying the origin of depression [11]. The role of monoamines in depression is widely studied. Particularly, 5-HT is highly connected with depression. This monoamine is produced from the amino acid Trp, and the disrupted activity of 5-HT pathways is related to the pathophysiology of depression [6]. Indeed, nowadays, antidepressants based on 5-HT reuptake to the synaptic cleft (SSRIs—selective 5-HT reuptake inhibitors) and are effective and one of the most prescribed worldwide, highlighting the role of 5-HT in depression. Nevertheless, it is important to keep in mind that this disease is extraordinarily complex, and impaired 5-HT activity can cause depression, but is neither necessary nor sufficient, with the presence of other factors being important [6], as referred above (Figure 2).

## 3. Trp Metabolism

**Figure 3:**
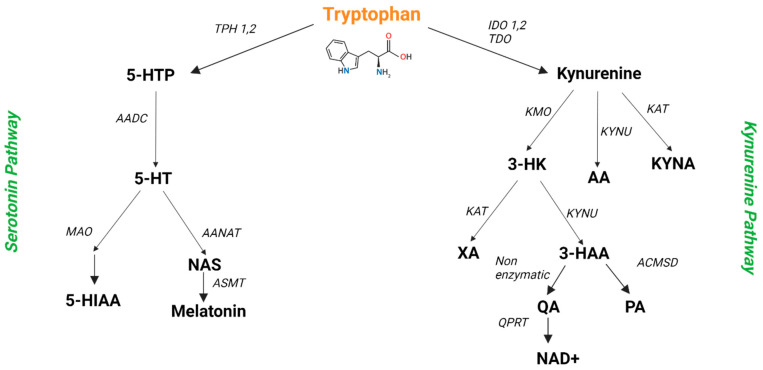
Summary of the two major metabolic pathways of the amino acid Trp: 5-HT and Kyn pathways. All of the abbreviations are described in text. Illustration created with BioRender [8].

Trp is an amino acid obtained exclusively through the diet. This amino acid is essential for several human processes, including gastrointestinal and nervous functions, being used for protein synthesis and metabolized essentially by two pathways: the Kyn and the 5-HT pathways. Additionally, in the gut, the microbiota metabolizes Trp by the indole pathway. Figure 3 summarizes the Trp metabolism by the Kyn and 5-HT pathway, which are the focus of this article.

The Kyn is the major Trp metabolism pathway (about 95% of free Trp is a substrate for this pathway) [12]. This pathway is important for physiological processes including the generation of cell energy, inflammation, immune response, and neurotransmission. Indeed, this metabolic pathway influences physical exercise responses and mental health, being linked to diseases such as depression, schizophrenia, cancer, and diabetes [4,5,12]. This pathway exists in several cells/tissues, such as the liver, brain, and immune cells [4]. The rate-limiting and first step in the Kyn pathway is the conversion of Trp to Kyn by the enzymes indoleamine 2-3-dioxygenase 1 and 2 (IDO1 and IDO2) and tryptophan 2,3-dioxygenase (TDO). Then, Kyn is mainly converted to 3-hydroxykyn (3-HK) by the enzyme kynurenine 3-monooxygenase (KMO). 3-HK is then converted to xanthurenic acid (XA) by the enzyme kynurenine aminotransferase (KAT) and to 3-hydroxyanthranilic acid (3-HAA) by the enzyme kynureninase (Kynu). Then, 3-HAA is metabolized in picolinic acid (PA) by aminocarboxymuconate-semialdehyde decarboxylase (ACMSD) and in quinolinic acid (QA) by non-enzymatic conversion. After that, QA is metabolized to NAD+ by the action of quinolinate phosphoribosyl transferase (QPRT). Additionally, to a lesser extent, Kyn is metabolized into kynurenic acid (Kyna) by the enzymatic action of KAT and in anthranilic acid (AA) by Kynu’s action [4,13].

The 5-HT pathway is also extremely important to human physiology. Serotonergic networks are crucial in behavioral aspects (such as mood, sexuality, memory, appetite, stress response, and anger), as well as other central nervous system effects (like motor control, regulation of circadian rhythm, and body temperature) and effects outside of the central nervous system, such as gastrointestinal regulation, nociception, mammary gland development, vasoconstriction/dilation, regulation of heart rate, and platelet aggregation [7]. This pathway’s first and rate-limiting step is the conversion of Trp to 5-hydroxytryptophan (5-HTP) by the enzymes tryptophan hydroxylase 1 or 2 (TPH1 or 2, mostly expressed in peripheral and in the central nervous tissues, respectively). Then, 5-HTP is decarboxylated by the aromatic acid decarboxylase (AADC) to form 5-HT, which can be metabolized to form N-acetylserotonin (NAS) by arylalkylamine N-acetyltransferase (AANAT), and then by N-acetylserotonin O-methyltransferase (ASMT) to form melatonin. 5-HT can also be metabolized by the enzyme monoamine oxidase (MAO) to form 5-hydroxyindoleacetic acid (5-HIAA), the major 5-HT metabolite [3,13,14].

Lastly, the indole pathway is conducted by the gut microbiota. Briefly, this pathway generates several metabolites (like indole and tryptamine) that are important to the host physiology. Indeed, these metabolites participate in processes such as immune system regulation, gastrointestinal motility, inflammation, and anti-oxidative effects [13,15].

## 4. Trp Metabolism in Depression—An Overlook

The Trp metabolism is widely involved in depression and other neuropsychiatric disorders, mainly by being responsible for the synthesis of both 5-HT and Kyn, which are important neuroactive compounds [3]. Indeed, a study focused on the effect of dietary Trp on affective disorders highlighted that a diet enriched in Trp may result in less depressive symptoms and better mood states of individuals. Additionally, a diet with low levels of Trp resulted in irritability and anxiety in comparison with when the same individuals had a Trp-rich diet [16].

Particularly, the role of 5-HT in this disease is deeply studied. Focusing on the metabolism of Trp to 5-HT/melatonin/5-HIAA, several aspects are intimately connected with depression’s etiology and pathophysiology. The enzyme TPH has an essential role in many mental disorders, including depression. Studies demonstrate that stress inhibits the expression of this enzyme, reducing the levels of 5-HT [17]. Indeed, studies also correlate the expression of TPH1 enzyme with depression and responses to antidepressant medication. A solid hypothesis is that the periphery cells that produce 5-HT have a TPH1 dysfunction, leading to deficient brain 5-HT levels. This leads to a homeostatic response to low 5-HT levels by the TPH2 enzyme, which was overexpressed in individuals that have committed suicide [18,19]. Additionally, both 5-HTP and 5-HT play important roles in depression. 5-HTP may help increase 5-HT levels, reducing the symptoms of depression. Evidence points out that the transport of 5-HTP across to the brain is deficient in depression [20,21]. Another study showed that the combination of 5-HTP with nialamide (an antidepressant, MAO inhibitor) was beneficial when compared with the administration of nialamide alone [20,22]. The same happened with the combination of L-deprenil (another MAO inhibitor) with 5-HTP [20,23]. Decreased levels of 5-HT and disruptions in its receptors and pathways are also widely associated with this pathology. Indeed, deficits in serotonergic transmission, including reductions in 5-HT neurons and their projections, are associated with this disease and failure of antidepressant responses. Additionally, the most commonly prescribed antidepressants are based on the blockade of 5-HT transporter (5-HTT or SERT), increasing the levels of 5-HT on the synaptic cleft, promoting antidepressant responses [6,24]. MAO enzyme imbalances are also associated with the pathology of depression. Indeed, monoamine oxidase inhibitors (MAOIs) have proven efficacy in treating depression [25]. MAO-A is more involved in the pathophysiology of depression, as elevated MAO-A activity and expression are observed in depressed individuals and in animal models of depression [26]. Nevertheless, MAO-B activity is also altered in depression. Indeed, a study revealed that elevated MAO-B levels were found in the prefrontal cortex of patients with major depressive episodes [27]. Other important metabolites in the 5-HT pathway are 5-HIAA and melatonin. Starting with 5-HIAA, a study reported that low levels of this metabolite in cerebrospinal fluid were associated with suicidal attempts in depressed individuals [28], being linked with low levels of 5-HT. Lastly, low levels of melatonin were also observed in depressed patients [29], despite some literature inconsistencies. For example, there are studies that correlate lower nocturnal serum/saliva levels of melatonin to depression, whereas others found no differences or even elevated levels (versus non depressed individuals) [30,31]. Nevertheless, studies in animals demonstrated that melatonin reduced neuroinflammation induced by lipopolysaccharide (LPS), as well as NF-κB expression in the cortex and the hippocampus of these animals, leading to the attenuation of autophagy impairment and improvement of depressive symptoms [32].

The Kyn pathway is also implicated in depression. Indeed, this pathway is strongly activated in depression, and may contribute to the progression of the disease [33]. Both IDO and TDO enzymes are overactivated during depression, highlighting the use of IDO and TDO inhibitors as potential drugs to treat depressive disorder [33]. Additionally, it is important to remember that the metabolite Kyna is considered a neuroprotective compound, whereas 3HK is neurotoxic. In depression, there is an imbalance between these compounds. Indeed, astrocytes mainly produce Kyna because they lack the enzyme KMO, whereas microglia and macrophages produce essentially the neurotoxin QA from the 3HK pathway [19,34,35]. In depression, it is stated that there is a loss of astrocytes, contributing to the overactivation of 3HK pathway. In turn, the overactivation of this pathway induces even more astrocyte and neuronal apoptosis, lowering the levels of important neuroprotective factors produced by these cells, such as glial-derived neurotrophic factor (GDNF) [19]. Thus, KMO activation relates to depression. Additionally, it was observed that increases in the levels of QA contribute to reduced levels of dopamine, choline, and γ-aminobutyric acid (GABA) [36], as well as disturbances in glutamatergic pathways, also known to be dysfunctional in depression [19,37]. Furthermore, in depression, hippocampal atrophy is described. This hippocampal atrophy may also be linked with imbalances in neurotoxic/neuroprotective compounds such as QA/Kyna [38]. Evidence also suggests that the overproduced proinflammatory cytokines in depression induce the IDO enzyme, promoting the Kyn pathway, and thus decreasing the activation of the 5-HT pathway and reducing 5-HT production [38]. In mice exposed to chronic unpredictable mild stress, a Trp-rich diet shifted the Trp metabolism more toward the 5-HT pathway than to the Kyn pathway, which was enhanced in these mice previously to Trp supplementation [39]. Thus, several pieces of evidence indicate that the Kyn pathway is an important player in depression, being a potential therapeutic target. In this review, we will focus on processes such as inflammation and neurotrophic factor expression in depression, associated with Trp metabolism in the Kyn and 5-HT pathways.

### 4.1. Trp Metabolism and Depression’s Associated Neuroinflammation

Neuroinflammation is a hallmark widely associated with several neurological and neuropsychiatric diseases, such as depression and Alzheimer’s disease. By regulating the production of immune factors and immune cell activation, neuroinflammation assumes an important role in depression [40]. There is a positive relationship between antidepressant therapy/psychotherapy and the reduction of inflammatory signs, highlighting the connection between depression and inflammatory processes [41]. Proinflammatory cytokines are substances produced essentially by activated macrophages, involved in the overactivation of inflammatory reactions. Some examples are IL-1β, IL-6, and TNF-α [42]. These cytokines are involved in HPA–axis overactivation, leading to an increase in cortisol concentration and glucocorticoid receptor resistance, mechanisms linked to the pathogenesis of depression [41,43].

Alteration of Trp metabolism by proinflammatory cytokines is also an important process that links inflammation to depression. Indeed, these cytokines induce the IDO enzyme, promoting the activation of the Kyn pathway and consequently reducing the activation of 5-HT pathway of Trp, leading to an overall decrease in 5-HT synthesis [38]. Additionally, as referred above, the overactivation of Kyn pathway leads to an imbalance in neurotoxic/neuroprotective compounds, particularly 3-HK/QA/Kyna. Studies revealed that patients treated with IFN-α have reduced levels of Trp, increased levels of Kyn and Kyn/Trp ratio activity, and increases in depressive symptoms [44]. Other evidence that highlights the importance of Kyn pathway in inflammatory processes associated with depression is the efficacy of ketamine as an antidepressant agent. Indeed, this drug appears to induce anti-inflammatory effects. Animal studies and clinical evidence revealed that this drug decreased pro-inflammatory cytokines levels (such as IL-6 and TNF-α), decreased levels of IDO enzyme, and reduced the activation of the neurotoxic pathway of the Kyn pathway [45,46,47,48,49]. Furthermore, COVID-19-associated depression is widely reported [50]. A recent study also indicated that, in COVID-19 patients, there was a major urinary increase in Kyn, which was associated with disease severity and systemic inflammation [51] and may be associated with the high levels of depressive symptoms in COVID-19 patients. Indeed, inflammatory cytokines released in the process of COVID-19 inflammation activate both the HPA–axis and the IDO enzyme, increasing the levels of toxic metabolites of the Kyn pathway, which increases overall neuroinflammation and neuronal death, promoting depressive conditions [52]. Additionally, antidepressants with anti-inflammatory properties inhibit IDO induction by decreasing the levels of proinflammatory cytokines in immune-activated individuals, contributing to attenuation of depressive symptoms [53]. The role of the immune system in depression is also supported by the homeostasis of glutamatergic neurotransmission, which can be regulated by the QA/Kyna ratio, synthetized by microglia and astrocytes, respectively [54]. Overactivation of the neurotoxic arm of Kyn pathway was also observed in suicide attempters, coupled to increased inflammatory responses. By raising the levels of QA (agonist of NMDA-receptor—N-methyl-D-aspartate receptor, a glutamate receptor), NMDA-receptor was overactivated, supporting the use of NMDA-receptor antagonists such as ketamine and dextromethorphan in the treatment of this disease [55]. Another recent study also supported the role of Kyn in depression’s associated neuroinflammation. Saffron administration in mice attenuated inflammation-related metabolic pathways and modulated the expression of Kyn-related neurotoxicity, attenuating depressive-like behaviors in these animals [56]. Furthermore, the administration of the anti-inflammatory herb Radix Polygalae to mice exposed to chronic restraint stress led to modulation of inflammation, microbiota, and Trp/Kyn metabolism. Indeed, Radix Polygalae reversed the 5-HIAA decrease and the Trp, Kyna, and 3-HK increase induced by the stress exposure. This herb increased the 5-HT/Kyn ratio, which was decreased under the stress exposure, revealing an interaction between the anti-inflammatory mechanism of action and Trp metabolism [57]. Another study that evaluated the link between Trp metabolism and depressive symptoms in obesity reported that elevated levels of C-reactive protein (that reflects inflammation) correlated with low levels of Trp and Trp indole pathway metabolites such as indole-3-carboxaldehyde, observed in the more severe cases of depressive behavior [58]. In post-partum women with severe depression, dysregulation of the immune response and Kyn pathway, as well as a reduction in 5-HT levels, was also observed. Indeed, Trp was directed more towards the Kyn pathway than to the 5-HT pathway, and had high levels of inflammatory markers, such as IL-6 and IL-8 [59].

Inflammation is also connected to disturbances in the 5-HT pathway of Trp metabolism, not only by the overactivation of Kyn pathway (explained above), but also by other mechanisms. There is a large amount of evidence showing that, in patients with depression, proinflammatory cytokines originated by the microglia (TNF-α for example) reduce the presence of 5-HT [60]. Indeed, modulation of 5-HT levels by the administration of SSRIs reduced the Th17/Treg ratio, supporting an anti-inflammatory action of these drugs [61]. Other studies also demonstrated this connection between 5-HT and inflammatory processes. Mice treated with rapamycin (blocks activation of immune T and B cells) showed increased levels of 5-HT (compared with healthy controls) [62]. Another depressogenic action of cytokines is the increased expression of the 5-HT transporter, SERT [63]. This transporter was reported to be upregulated in neurons by the cytokines IL-1β and TNFα. Indeed, it was found that p38 mitogen-activated protein kinase induced activation of SERT by inflammatory cytokines [64]. Additionally, SERT expression in immune cells was also observed in response to IFNα [65]. Other studies also support this evidence of SERT overexpression by the action of cytokines. After cytokine-induced LPS administration in mice, SERT activity was stimulated, triggering depressive-like behavior in these animals. In cultured serotonergic cells and nerve terminal preparations in vitro, SERT activity was also upregulated by inflammatory cytokines [66]. Cytokines are also reported to affect the synthesis of monoamines such as 5-HT through disruption of tetrahydrobiopterin, an important enzyme co-factor to Trp hydroxylase [67,68]. Additionally, high stress levels can also lead to increased levels of pro-inflammatory cytokine production, leading to changes in serotonergic pathways, involved in the development of depression [67]. Paroxetine, an SSRI, has also been demonstrated to be an effective treatment for minimizing depression induced by IFNα, in patients with melanoma and hepatitis C [69,70]. These studies support the interplay between neuroinflammation and Kyn/5-HT pathways in depressive disorder.

### 4.2. Trp Metabolism and Depression’s Associated Chronic Stress

Uncontrolled stress induces changes in the central nervous system, promoting the development of neuropsychiatric disorders such as depression [41]. The human stress response is strictly regulated by the HPA–axis, which, when activated, is responsible for the increase in glucocorticoid levels, particularly cortisol, an important player in depression and other neurological disorders [71].

As discussed above, proinflammatory cytokines promote an imbalance in neurotoxic/neuroprotective metabolites by the overactivation of the neurotoxic arm of the Kyn pathway. Besides that, these proinflammatory cytokines also activate the HPA–axis, leading to stress responses that underlie depressive states, such as the observed hippocampal atrophy [38]. Particularly, by activating NMDA receptors, QA stimulates the production of interleukins such as IL-6, promoting the overactivity of this axis [72]. Elevated levels of cortisol correlate with lower levels of Trp in the plasma and a higher Kyn/Trp ratio, observed in patients who tried to commit suicide [73]. Indeed, cortisol is known to activate TDO, increasing Kyn production and shifting Trp metabolism from 5-HT to Kyn production [74]. Thus, the conversion of Trp in Kyn is promoted by chronic stress levels, mainly by elevations in cortisol and pro-inflammatory cytokines, which in turn activate IDO/TDO enzymes [75]. Indeed, treatment with allopurinol (TDO inhibitor) prevented stress-related reduction in brain 5-HT concentrations by blocking TDO activity. This treatment led to a reduction in the Kyn pathway activation ratio [76,77]. Additionally, the administration of 1-methyl-Trp (an inhibitor of IDO enzyme) alleviated depressive-like behavior in rodents exposed to LPS-induced stress [78]. Indeed, LPS exposure increased brains’ IDO mRNA expression in rodents, resulting in overactivation of the Kyn pathway [79]. Physical exercise also supports the effects of Kyn pathway in depression’s associated stress. The skeletal muscle PGC-1α1 enzyme’s activity is induced by physical exercise, inducing kat expression and the conversion of Kyn into Kyna, a neuroprotective metabolite of the Kyn pathway. This conversion of Kyn into Kyna controls the Kyn/Kyna balance, reducing the levels of free Kyn and protecting the brain against stress-induced depressive behaviors [80]. PGC-1α1 activity is known to reduce with age and with pathologies such as diabetes. Thus, this lack of activity may contribute to the depression associated with other pathologies/age [76].

Serotonergic networks are deeply influenced by stress responses. Indeed, some experiments demonstrate this connection. For example, a study carried out in cynomolgus monkeys revealed that the animals that were more sensitive to stress underexpressed genes such as TPH2 and 5-HT 1A receptor, important genes in the normal functioning of 5-HT pathways [81]. Additionally, as referred above, it was revealed that rats exposed to stress presented lower levels of 5-HT compared with healthy or treated rats. TPH1 and TPH2 were also less expressed in the rats exposed to high stress levels [17]. Other studies support the connection of cortisol to serotonergic pathways. For example, the administration of crocin decreased cortisol levels and increased the levels of 5-HT, ameliorating depressive-like behavior in mice [82]. The same profile of response was also obtained with the administration of gossypetin [83], aqueous extracts of miswak, and date palm [84]. Lower levels of HPA–axis hormones and increased levels of 5-HT and brain-derived neurotrophic factor were also obtained with Trp oligopeptide diets, promoting positive effects on anxious depression in mice [85]. Taken together, all of this information highlights the relationship between Trp metabolism and the stress response present in depression.

### 4.3. Trp Metabolism and Microbiota in Depression

A lot of studies support the role of gut microbiota in the pathogenesis of disorders such as depression. Indeed, the changes in the gut microbiota observed in depression affect the HPA–axis, neurotransmitter levels, and inflammatory processes [86]. The use of germ-free models to study the relationship between depression and the microbiome has revealed the crucial role of these microorganisms in normal brain functioning. For example, exaggerated levels of corticosterone and adrenocorticotropin (HPA–axis hormones) were observed in germ-free mice exposed to elevated levels of stress [87]. Furthermore, the administration of *Lactobacillus* sp. normalized the elevated corticosterone levels present in rats after maternal separation [88].

A study that involved a murine model of chronic restraint stress revealed that these animals had depressive-like behavior, as well as strong activation of the Kyn pathway. Indeed, IDO was overactivated in the brain and the gut. In these animals, the microbiome profile was altered, and the treatment with Parabacteroides elevated the 5-HT concentration, supporting the connection between 5-HT/Kyn pathways and the microbiome [89]. Another study revealed that, in stressed mice, there were reduced levels of *Lactobacillus* and high levels of Kyn, reflected in behavioral alterations. In these mice, when the *Lactobacillus* population was restored, Kyn metabolism was suppressed by IDO1 inhibition in the intestine, particularly by the reactive oxygen species produced by these microorganisms [90]. The evaluation of the antidepressant activity of probiotics such as *Bifidobacteria* infantis in rats also revealed that, compared with controls, these rats had a marked increase in plasma concentrations of Trp and Kyna, as well as reduced 5-HIAA levels, especially in the frontal cortex [91]. In depressive mice, a Trp-rich diet restructured the gut microbiome, increasing the number of *Lachnospiracea*, *Lactobacillus*, and *Bifidobacterium* [39].

The direct influence of gut microbiota in serotonergic networks is known. A study showed that oral administration of *S. boulardii* attenuated the LPS-induced depressive behaviors, increasing the levels of brain-derived neurotrophic factor and 5-HT in the serum [92]. Another study involved rats also exposed to stress, particularly chronic unpredictable mild stress. These rats developed depressive-like behavior and their fecal microbiota were evaluated by 16S rRNA sequence analysis. The results revealed that the microbiota of these rats differed significantly from healthy controls, which may contribute to depressive-like behavior by interfering with Trp metabolism. Indeed, the levels of 5-HT and TPH2 were low in the brain, contrasting with high levels of IDO expression [93]. The comparison of germ-free and conventional animals also indicated that the plasma levels of 5-HT in conventional animals were 2.8-fold higher than in germ-free animals, supporting the role of microorganisms in 5-HT production [94]. In another study, *L. lactis* strain WHH2078 increased 5-HTP levels and the expression of TPH1 in cells. In mice exposed to chronic unpredictable mild stress, these bacteria also alleviated the depressive-like behaviors, restoring central 5-HT and 5-HTP levels [95]. Another study evaluated the effect of a mung bean protein diet in undernourished rats. This diet led to the reproduction of probiotics, particularly *Bifidobacteria* and *Lactobacillus*, as well as increased levels of Trp and 5-HTP in the serum, compared with rats treated with low levels of mung bean protein, revealing low levels of cognitive dysfunction [96]. The administration of the probiotic strain *L. rhamnosus* IMC 501 to zebrafish also led to increased expression levels of bdnf and genes involved in the brain’s 5-HT signaling metabolism, particularly h1a, tph1b, tph2, htr1aa, slc6a4a, and mao. Indeed, this was correlated with the behavior of the fishes, particularly the shoaling behavior. Additionally, a significant increase in *Firmicutes* was also observed [97]. These studies support the role of the gut microbiota in Trp metabolism associated with depressive disorder.

### 4.4. Trp Metabolism and Brain-Derived Neurotrophic Factor Expression in Depression

Different neurotrophic factors are highly connected to depression, notably BDNF. These factors are important to neuronal plasticity, a process defined by the adaptation of the nervous system in response to different stimuli. Depressed individuals have decreased levels of BDNF in the blood and brain structures connected with depression, such as the hippocampus. Additionally, antidepressants such as SSRIs increase BDNF expression [98,99]. Trp metabolism is implicated in BDNF function. Indeed, a study reported that the depletion of Trp in healthy patients led to a compensatory increase in serum BDNF levels. This response was not observed in depressed individuals, in which BDNF levels, as well as plasma Trp levels, remained low [100]. In another study in depressive mice exposed to chronic unpredictable chronic stress, Trp supplementation also improved the expression of BDNF [39].

Kyn pathway and BDNF expression are also connected in the context of depression. By interacting with the NMDA receptor, QA induces signaling pathways that reduce BDNF expression [45]. Other Kyn neurotoxic metabolites besides QA also weaken glial-neuronal networks important to neurotrophic factor synthesis, particularly BDNF [19]. Indeed, studies demonstrate that BDNF may modulate the Kyn pathway. After exposure to stress conditions, heterozygous mice (BDNF+/−, about 50% reduction of BDNF expression) showed increased activation in the neurotoxic arm of the Kyn pathway, increasing the level of neurotoxic metabolites such as 3-HK, in contrast with the wild-type animals [101]. In another study, the Kyn pathway was altered in mice displaying BDNF Val66Met polymorphism. This polymorphism relates to increased predisposition to develop psychiatric disorders. In this study, these mice showed overactivation of the Kyn pathway [102]. Another study evaluated the effect of the acute examination stress in healthy students, revealing that the elevation of BDNF levels present in these students limited the neuroinflammatory arm of the Kyn pathway, supporting an interplay between BDNF and the Kyn pathway [103]. This interplay was also supported by a study that revealed that blockade of IDO1 attenuated depressive-like behavior in mice exposed to chronic unpredictable mild stress, with a concomitant increase in hippocampal BDNF expression and neurogenesis in the hippocampus [104].

Impaired expression of BDNF alters 5-HT pathways. For example, it is known that BDNF stimulates the plasticity of 5-HTergic neurons [105]. This neurotrophic factor reduces SERT and 5-HT1A receptor function mainly in the hippocampus, and reduces 5-HT2A receptor function in other brain areas such as the prefrontal cortex [106]. Indeed, studies in a rat model of acute psychological stress revealed that agonists of 5-HT1A and 5-HT2A receptors increased BDNF protein expression in diverse brain regions, opposing the effects of 5-HT1A and 5-HT2A receptor antagonists, supporting the connection between BDNF and the 5-HT system [107]. In raphe neurons, BDNF promoted the expression of TPH and upregulated the uptake of 5-HT. This neurotrophic factor also promoted the development and function of serotonergic neurons [108]. Indeed, the connection of BDNF to serotonergic pathways is supported by the interaction of this factor with serotonergic receptors such as 5-HT1A and 5-HT2A, which were impaired in conditions when the BDNF gene was deleted [108,109,110]. Another piece of evidence that supports this connection is that the increased levels of 5-HT may increase BDNF levels, as observed upon administration of SSRI antidepressants. Indeed, the block of SERT enhances 5-HT pathways through the interaction with different 5-HT receptors that, in turn, increase CREB phosphorylation, which leads to increased levels of BDNF transcription [108]. Another interesting study evaluated the effects of gardening in elderly people. In the gardening group, Trp metabolism was increased, correlated with increased levels of BDNF [111]. Another recent study highlights the relationship between TPH2 expression and BDNF levels. The administration of a pargyline (MAO inhibitor) in zebrafish treated with an irreversible inhibitor of TPH2 (p-chlorophenylalanine) led to reduced BDNF levels, revealing an interdependence between 5-HT and BDNF systems in the antidepressant response [112]. The interaction between 5-HT neurotransmission and the BDNF-related pathways was also supported by another study. In this study, enhanced Trp intake led to increased activation of 5-HT pathways that, in turn, modulated the BDNF system, protecting against cognitive decline in aged rats [113]. Physical exercise is also known to upregulate the BDNF–5-HT system. Indeed, 5-HT participates in BDNF-mediated neuroplasticity, stimulated by aerobic physical exercise in rats [114]. Altogether, these studies support the interplay between BDNF and Trp metabolism in the 5-HT and Kyn pathways.

### 4.5. Pharmacological Modulation of Trp Metabolism in Depression—An Overlook

As described throughout this manuscript, Trp metabolism is crucial in the pathophysiology of depression, with great emphasis on the 5-HT pathway. Thus, the pharmacological modulation of the effects of this pathway plays a major role in the therapy of this disease. Nevertheless, it is important to refer that, in the management of depression, psychotherapy also assumes a heavy role. Table 1 summarizes the main drug classes related to 5-HT in the treatment of depression. These drugs act mainly downstream the production of 5-HT.

Therapies regarding the direct manipulation of Kyn pathways are still not available in the common medical practice for depression. However, future and intensive research may generate novel insight into this pathway and the way to manipulate it. Nevertheless, administration of 4-chlorokynurenine (an investigational antidepressant drug) enhanced the neuroprotective arm of the Kyn pathway, decreasing the neurotoxic arm [121]. Additionally, the administration of escitalopram revealed a 50% decrease in plasma QA, suggesting that these drugs reduce the neurotoxic arm of the Kyn pathway [122]. Chronic administration of antidepressants (amitriptyline, imipramine, fluoxetine, and citalopram) in rats also revealed an increase in Kyna production in the hippocampus and cortex, increasing the neuroprotective arm of the Kyn pathway [123]. Ketamine also led to increased levels of Kyna in the serum, associated with a reduction in depression severity [49]. IDO1, TDO, KMO, and KAT inhibitors are also under investigation, mainly for cancer and not depression purposes [124]. Indeed, investigations focusing on Trp metabolism may generate a next step on the treatment of depression.

## 5. Conclusions

It is extremely important to deeply study depression’s molecular mechanisms, aiming to understand this extremely complex disease that is very prevalent worldwide. New therapies urgently need to be developed, and are only possible when research efforts are present. The study of Trp metabolism in the context of depression may provide new knowledge and therapeutic possibilities. This amino acid, by being metabolized into the 5-HT or Kyn pathway, assumes a crucial role in several aspects profoundly connected to depression’s physiopathology, such as neuroinflammation, chronic stress, dysregulation in the gut microbiota, and BDNF expression levels. The maintenance of the Trp-Kyn/Trp-5-HT balance is critical for physiological homeostasis, being important to prevent the development of neuropsychiatric diseases such as depression.

## Figures and Tables

**Table 1:** Principal drug classes related to 5-HT in the treatment of depression.

Drug Class	Brief Description	Examples
SSRIs—Selective 5-HT Reuptake Inhibitors	Inhibit SERT at the presynaptic axon terminal, increasing the amount of 5-HT in the synaptic cleft [115]	Fluoxetine, sertraline, escitalopram, paroxetine [115]
MAOIs—Monoamine Oxidase Inhibitors	Block MAO enzyme, inhibiting the breakdown of 5-HT and other neurotransmitters, increasing their levels [116]	Moclobemide, tranylcypromine, phenelzine, isocarboxazid [116]
SNRIs—Serotonin–Noradrenaline Reuptake Inhibitors	Inhibit the reuptake of both 5-HT and norepinephrine, by blocking reuptake transporters, increasing their amount in the synaptic cleft [117]	Venlafaxine, duloxetine, desvenlafaxine [118]
TCAs—Tricyclic Antidepressants	Block the reuptake of 5-HT and norepinephrine, act as antagonists on post-synaptic cholinergic (alpha1 and alpha2), muscarinic, and histaminergic receptors (H1), enhancing neurotransmission [119]	Amitriptyline, imipramine, desipramine, clomipramine [118]
NaSSAs—Noradrenergic and Specific Serotonergic Antidepressants	Antagonism of 5-HT2 (5-HT2A and 5-HT2C) and 5-HT3 receptors, block α2 receptors, enhancing neurotransmission [120]	Mirtazapine [120]

## Data Availability

Not applicable.
